# Early and late recurrence after hepatectomy in patients with low-level HBV-DNA hepatocellular carcinoma under antiviral therapy

**DOI:** 10.1186/s13027-022-00468-6

**Published:** 2022-11-17

**Authors:** Ziqiang Li, Chengpeng Tan, Xiaohong Liu, Zhe Feng, Kun Li

**Affiliations:** Department of Hepatobiliary and Pancreatic Surgery, Hubei Provincial Clinical Medicine Research Center for Minimally Invasive Diagnosis and Treatment of Hepatobiliary and Pancreatic Diseases, Zhongnan Hospital of Wuhan University, No. 169, Donghu Road, Wuchang District, Wuhan, 430061 China

**Keywords:** Antiviral therapy, Recurrence, Hepatitis B virus, Low-level viremia, Hepatocellular carcinoma

## Abstract

**Background:**

Antiviral therapy has been shown to benefit long-term survival after curative hepatectomy in patients with hepatitis B virus (HBV)-associated hepatocellular carcinoma (HCC) with high levels of HBV-DNA, but the impact of antiviral therapy on patient recurrence in patients with low levels of HBV-DNA remains less clear.

**Methods:**

This was a retrospective cohort study analyzing 296 patients with HBV-associated HCC with HBV-DNA levels < 2000 IU/mL who underwent hepatectomy at Zhongnan Hospital of Wuhan University between March 2013 and December 2017, of whom 157 patients received antiviral therapy (antiviral group) and 139 patients did not receive antiviral therapy (non-antiviral group), propensity score matching was used for survival analysis of patients in both groups, and subgroup analysis of major risk factors was performed.

**Results:**

The baseline characteristics of the two groups were comparable. At a median follow-up of 54 months, the 1-, 3-, and 5-year overall survival rates after propensity score matching (PSM) were 94.9%, 80.8%, 66.5%, and 90.9%, 64.6%, 49.4% for the antiviral and non-antiviral groups, respectively, *p* = 0.009, and the corresponding 1-, 3-, and 5-year RFS for the two groups were 81.8%, 76.8%, 76.8%, and 67.7%, 55.6%, 55.6%, respectively. *p* = 0.001, and the overall survival and recurrence-free survival were significantly better in the antiviral group than in the non-antiviral group. Multi-factor COX regression analysis showed that prothrombin time ≥ 13 s, methemoglobin level ≥ 20 ng/ml, platelet count ≥ 100 × 10^9^/L, tumor size > 5 cm, tumor multiplicity was associated with early recurrence, and antiviral treatment was an independent protective factor for early recurrence of HCC (HR, 0.431; 95% CI 0.274–0.679; *p* < 0.001), but not associated with a low risk of late relapse (HR, 0.822; 95% CI 0.526–1.284; *p* = 0.389), and the main risk factors for late relapse included AST levels > 40 IU/ml, ALP levels > 130 IU/L, and the presence of satellite nodules, and subgroup analysis showed that compared to HBeAg-positive patients, antiviral therapy could significantly prolonged the recurrence-free survival of HBeAg-negative patients.

**Conclusion:**

Antiviral therapy reduces early tumor recurrence after hepatectomy in patients with low levels of HBV-DNA.

## Background

Hepatocellular carcinoma is the fourth most common malignancy worldwide with a high mortality rate, ranking third among the causes of cancer-related deaths worldwide [1, 2]. Current treatments for hepatocellular carcinoma include surgical resection, chemotherapy, radiofrequency ablation, transcatheter arterial chemoembolization, and liver transplantation, among which radical hepatectomy is still the main treatment for HCC [3, 4]. However, even after effective treatment, the recurrence rate of hepatocellular carcinoma remains high, with a 5-year recurrence rate of approximately 70% [5]. HBV infection is considered the most important causative agent of HCC, with approximately 240 million people infected worldwide, especially in the Asia-Pacific and African regions [6–8]. Related studies have shown that a high HBV-DNA viral load may be a major risk factor for relapse [9–11]. Several recent studies have shown that HBV viral replication levels play an important role in the development of HCC and tumor recurrence, that antiviral drugs (tenofovir disoproxil fumarate, entecavir) can be used to control HBV-DNA viral load, and that postoperative antiviral therapy with nucleotides/nucleoside analogs significantly improves patient survival after surgery [12–19]. These findings suggest that antiviral therapy may have a positive impact on reducing recurrence in patients with HBV-associated hepatocellular carcinoma. However, some studies have also shown no difference in prognosis of HBV-associated HCC patients with antiviral drug therapy [20, 21]. Overall, there are relatively few data regarding the effectiveness of antiviral therapy after curative hepatectomy for HCC, especially in patients with HBV-associated HCC with low levels of HBV-DNA, and it is still not well known whether antiviral therapy is effective in preventing recurrence of metastasis after curative hepatectomy in patients. To this end, we conducted a retrospective study to assess the effect of antiviral drug therapy on recurrence and survival after hepatectomy in patients with HBV-associated HCC with low levels of HBV-DNA.

## Methods

### Patients

Patients with HBV-related HCC treated with hepatectomy at Zhongnan Hospital of Wuhan University between March 2013 and December 2017 were identified using a prospectively collected database. Patients’ clinical information was obtained from the hospital’s electronic medical record system. The diagnosis of HCC was confirmed based on histopathological examination of postoperative liver specimens.

The inclusion criteria for patients were: (I) age ≥ 18 years; (II) initiation of antiviral drugs entecavir or tenofovir or lamivudine 3 months before or 3 months after hepatectomy; (III) postoperative liver histopathology confirmed hepatocellular carcinoma; (IV) positive hepatitis B surface antigen (HBsAg); (V) serum HBV-DNA level < 2000 IU/mL; and (VI) follow up for at least 3 months. Patients meeting any of the following criteria were excluded: (I) coexistence of other malignancies; (II) co-infection with hepatitis C virus or HIV; (III) preoperative radiofrequency ablation or other antitumor therapy; (IV) irregular use of the antiviral drugs entecavir or tenofovir; and (V) poor liver function (Child-Pugh class C).

All patients underwent preoperative imaging including chest radiograph, abdominal ultrasound, enhanced computed tomography CT of the abdomen to MRI, and laboratory tests including but not limited to HBV-DNA, HBsAg, HBeAg, platelet count, prothrombin time, serum albumin, serum total bilirubin, carcinoembryonic antigen, serum methemoglobin, aspartate aminotransferase, glutamate aminotransferase, etc. Pathologists scientifically evaluated the oncological features of the resected liver specimens.

### HCC diagnosis and recurrence

The preoperative criteria for the diagnosis of HCC were: (I) dynamic contrast-enhanced CT or magnetic resonance imaging showing typical features of HCC (i.e., nodules larger than 1 cm with arterial phase enhancement and portal or delayed phase washout) and/or (II) persistent increase in serum alpha-fetoprotein (AFP) levels. Curative liver resection was defined as the removal of all tumor tissue visible to the naked eye, with histopathological examination revealing no tumor cells at the margins of the residual liver and no residual tumor on postoperative imaging. Tumor recurrence should be considered when enhanced CT or MRI reveals new intrahepatic tumor lesions with typical imaging features consistent with HCC, and extrahepatic metastases should be highly suspected if new tumor lesions are found in organs other than the liver along with elevated AFP levels. Surgical indications for re-excision after recurrence are isolated or oligonodular tumors with sufficient residual liver tissue. Radiofrequency ablation, TACE or other combination therapy should be considered when re-excision cannot be performed.

### Follow-up

All patients were followed up for the first time in the first month after surgery, every 3 months in our outpatient clinic or local hospital for the first 2 years after surgery, and every 6 months thereafter, and were advised to continue antiviral treatment with TDF or ETV. The follow-up mainly included computed tomography, serum biochemical liver function, serum methemoglobin and HBV-DNA viral load measurement. If abnormal imaging or biochemical parameters are found, dynamic enhancement CT or magnetic resonance imaging should be performed immediately to confirm whether it is tumor recurrence. The primary outcome index is recurrence-free survival (RFS), defined as the time interval from the date of surgery to the date of detection of tumor recurrence, and the secondary outcome index is overall survival (OS), defined as the time interval from the date of surgery to the date of death of the patient from any cause or the date of the last follow-up visit. The index date was defined as the date of surgical resection for HCC. The total follow-up time for all patients ranged from 3 months to 70 months, with a median follow-up time of 54 months and a last follow-up date of February 10, 2022.

### Definitions

Early recurrence of hepatocellular carcinoma was defined as recurrence within 2 years after curative hepatectomy, while recurrence after 2 years of curative hepatectomy was defined as late recurrence [5].

### Statistical analysis

Continuous data are expressed as mean ± standard deviation, and non-normally distributed data are expressed as the median of the specific range. Parametric t-tests or Mann–Whitney nonparametric U-tests were used for comparison of continuous variables, whereas chi-square tests or Fisher exact tests were used to test categorical variables. Survival analysis was performed using the Kaplan–Meier method, and log-rank tests were used to compare differences. Cox proportional risk regression models were used to perform univariate and multifactorial analyses to identify risk factors for tumor recurrence and to make predictions about the factors associated with influencing RFS and OS. Subgroup analysis was used to analyze the major risk factors, and *p* < 0.05 was considered statistically significant. All statistical analyses were performed using the statistical software SPSS 25.0. Figures were made with GraphPad Prism 9 software.

## Results

### Clinical characteristics of study patients

A total of 428 patients with HBV-associated HCC were treated with curative hepatectomy at our institution between March 2013 and December 2017, 296 patients with serum HBV-DNA levels < 2000 IU/mL were included in the study, 157 (53.0%) of whom received antiviral therapy (entecavir 115, tenofovir 37, lamivudine 5), another 139 (47.0%) patients did not receive any form of antiviral therapy. Table 1 shows the baseline characteristics of the entire cohort, with a mean age of 56.5 years, mostly male patients (n = 242, 81.8%), and a Child–Pugh score of A in 291 (98.3%) except for 5 (1.7%) patients with B. The mean BCLC stage was 0 or A in 126 (42.6%) patients. The mean tumor size was 5.5 cm, and the presence of cirrhosis was confirmed by pathological histology in 196 (66.2%) patients, with higher AFP levels (*p* < 0.001), higher PLT levels (*p* = 0.026) and more patients with BCLC stage 0 or A (*p* = 0.005) in the antiviral treatment group compared with the non-antiviral group.

To reduce the impact of potential confounders between the two groups on OS and RFS comparisons, we performed propensity score matching, and among the 99 matched pairs of patients, the baseline characteristics of the antiviral and non-antiviral groups did not show significant differences and were considered to have achieved covariate balance (Table 2).

**1:** Baseline characteristics and clinical data

	Non-antiviral group, n = 139	Antival group, n = 157	*p* value
Age, years	56 (49–65)	57 (49–65)	0.722
Male sex, *n* (%)	115 (82.7)	127 (80.9)	0.682
ALT, IU/m	35 (25–54)	35 (26–51)	0.760
AST, IU/ml	37 (28–56)	40 (30–61.5)	0.494
ALP, IU/L	93 (75-140.5)	96 (73.5–119)	0.462
ALB, g/L	40.5 (36.9–43.2)	39.5 (36.0–42.6)	0.159
Total bilirubin, µmol/L	17.2 (14.1–25.0)	17.9 (13.6–23.5)	0.544
PT, s	11.4 (10.8–12.2)	11.5 (11.0–12.35)	0.236
AFP, ng/ml	8.1 (2.8-310.1)	156.3 (16.5–1038.8)	< 0.001
PLT, 10^9^/L	95 (88–183)	97 (92.5–207)	0.026
Satellite nodules, *n* (%)	17 (12.2)	29 (18.5)	0.139
Capsular, n (%)	107 (77.0)	126 (80.2)	0.492
Moderate/poor tumor differentiation, n (%)	54 (38.8)	57 (36.3)	0.652
Vascular invasion, n (%)	42 (30.2)	23 (14.6)	0.001
Tumor size, cm	6 (3.5–7.0)	5 (3–8)	0.199
Multiple tumor, *n* (%)	22 (15.8)	22 (14.0)	0.363
Cirrhosis, *n* (%)	95 (68.3)	101 (64.3)	0.466
Hypertension, *n* (%)	40 (28.8)	39 (24.8)	0.445
Diabetes mellitus, *n* (%)	26 (18.7)	26 (16.6)	0.628
History of smoking, n (%)	55 (39.6)	62 (39.5)	0.989
Alcohol abuse, n (%)	54 (38.8)	52 (33.1)	0.305
HBeAg positive, *n* (%)	27 (19.4)	24 (15.2)	0.347
Child–Pugh score			0.135
A	135	156	
B	4	1	
BCLC stage			0.005
Very early (0)	10	17	
Early (A)	38	61	
Intermediate (B)	48	56	
Advanced (C)	43	23	

**Table 2:** Baseline characteristics of the study patients after the 1:1 propensity score analysis

	Non-antiviral group, n = 100	Antiviral group, n = 100	*p* value
Age, years	56 (50–65)	59 (50–65)	0.539
Male sex, n (%)	83 (83)	83 (83)	> 0.999
ALT, IU/m	33.5 (24.0-46.8)	35 (27–56)	0.258
AST, IU/ml	34 (28–52)	39.0 (29.2–57.8)	0.382
ALP, IU/L	90.5 (75.0-129.8)	96.5 (73.2–128.8)	0.59
ALB, g/L	40.5 (37.0-43.2)	40.6 (37.0–43.0)	0.931
Total bilirubin, mg/dl	16.9 (14.2–22.4)	18.3 (12.9–24.1)	0.851
PT, s	11.7 (10.8–12.5)	11.4 (11.0–12.2)	0.479
AFP, ng/ml	11.9 (3.2-357.9)	34.5 (18.0–828.2)	0.002
PLT, 10^9^/L	96 (88–199)	97 (91–198.8)	0.287
Satellite nodules, n (%)	15 (15)	15 (15)	> 0.999
Capsular, n (%)	21 (21)	17 (17)	0.471
Moderate/poor tumor differentiation, n (%)	35 (35)	36 (36)	0.883
Vascular invasion, n (%)	19 (19)	13 (13)	0.247
Tumor size, cm	5.5 (3–7)	5 (3–8)	0.94
Multiple tumor, n (%)	15 (15)	9 (9)	0.342
Cirrhosis, n (%)	66 (66)	65 (65)	0.882
Hypertension, n (%)	26 (26)	29 (29)	0.635
Diabetes mellitus, n (%)	17 (17)	21 (21)	0.471
History of smoking, n (%)	38 (38)	40 (40)	0.772
History of drinking, n (%)	35 (35)	34 (34)	0.882
HBeAg positive, n (%)	17 (17)	18 (18)	0.852
Child–Pugh score			0.316
A	100	99	
B	0	1	
BCLC stage			0.693
Very early (0)	8	10	
Early (A)	35	37	
Intermediate (B)	38	40	
Advanced (C)	19	13	

### Predictors of OS and RFS

**Fig. 1:**
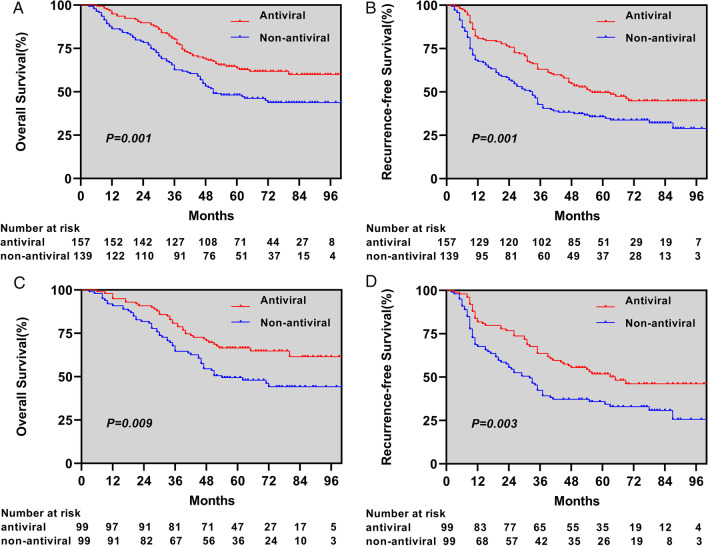
Comparison of overall survival (**A**) and recurrence-free survivals (**B**) between the two groups in the entire cohort and in the propensity score-matched cohort the overall survival (**C**) and recurrence-free survivals (**D**) between the two groups

The median follow-up time of patients was 54 months,174 (58.8%) patients developed tumor recurrence during the follow-up period, of which 95 (54.6%) had early recurrence, 79 (45.4%) had late recurrence, and 135 (45.6%) patients died.In the whole cohort, OS and RFS were significantly prolonged in the antiviral group compared to the non-antiviral group. The 1-, 3-, and 5-year OS was 94.9%, 80.3%, 63.7% and 86.3%, 62.6%, 48.2% in the antiviral and non-antiviral groups, respectively, *p* = 0.001, and the corresponding 1-, 3-, and 5-year RFS was 80.9%, 63.1%, 49.7% and 67.6%, 42.8%, 35.7% in the two groups, respectively, *p* = 0.001. In 198 patients (99 in the antiviral group and 99 in the non-antiviral group) after PSM, 67 (33.8%) experienced relapse (23 in the antiviral group and 44 in the non-antiviral group) and 88 (44.4%) experienced death (35 in the antiviral group and 53 in the non-antiviral group). There were significant differences in OS and RFS between the two groups, with the 1-, 3-, and 5-year OS was 94.9%, 80.8%, 66.5% and 90.9%, 64.6%, 49.4%, respectively, *p* = 0.009, and the corresponding 1-, 3-, and 5-year RFS was 81.8%, 76.8%, 76.8% and 67.7%, 55.6%, 55.6%, respectively, *p* = 0.001 for the two groups (Fig. 1A).

To identify factors associated with the impact on OS and RFS in patients after hepatectomy for HBV-related HCC, we performed univariate and multivariate COX regression analyses on the entire cohort and included parameters that had a significant impact on outcome in the univariate analysis in the multivariate analysis. Methemoglobin levels ≥ 20 ng/ml (HR, 2.227; 95% CI 1.513–3.278; *p* < 0.001), low to moderate tumor differentiation (HR, 1.625; 95% CI 1.150–2.297; p = 0.006), tumor size > 5 cm (HR, 2.237; 95% CI 1.130–4.429; *p* = 0.021), and tumor multiplicity (HR, 2.021; 95% CI 1.265–3.228; *p* = 0.003) were independent risk factors associated with OS. Independent risk factors affecting RFS included methemoglobin level ≥ 20 ng/ml (HR, 2.124; 95% CI 1.506–2.995; *p* < 0.001), alkaline phosphatase level > 130 IU/L (HR, 1.820; 95% CI 1.302–2.545; *p* < 0.001), tumor hypomedifferentiation (HR 1.462; 95% CI 1.067–2.002; *p* = 0.018), and tumor multiplicity (HR, 1.815; 95% CI 1.170–2.815; *p* = 0.008). Antiviral therapy was an independent prognostic factor not only for improving overall survival (HR, 0.500; 95% CI 0.343–0.728; *p* < 0.001) but also for RFS (HR, 0.529; 95% CI 0.377–0.741; *p* < 0.001) (Tables 3, 4).

**Table 3:** Univariate and multivariate analysis of overal survival

	Univariate analysis		Multivariate analysis	
	HR (95% CI)	*p* value	HR (95%CI)	*p* value
Age, years (> 60 vs ≤ 60)	1.030 (0.731–1.452)	0.865		
Gender (Male vs Female)	1.182 (0.749–1.867)	0.473		
ALT, IU/ml (> 40 vs ≤ 40)	0.768 (0.539–1.095)	0.144		
AST, IU/ml (> 40 vs ≤ 40)	1.252 (0.893–1.755)	0.192		
ALP, IU/L (> 130 vs ≤ 130)	1.144 (0.778–1.681)	0.494		
ALB, g/L (< 35 vs ≥ 35)	1.072 (0.690–1.667)	0.757		
Total bilirubin, µmol/L (> 17 vs ≤ 17)	1.249 (0.889–1.755)	0.200		
PT, s (≥ 13 vs < 13)	1.763 (1.132–2.745)	0.012		
AFP, ng/ml (≥ 20 vs < 20)	1.919 (1.343–2.742)	< 0.001	2.227 (1.513–3.278)	< 0.001
PLT, 10^9^/L (≥ 100 vs < 100)	1.251 (0.864–1.810)	0.236		
Satellite nodules (yes vs no)	1.741 (1.154–2.627)	0.008		
Capsular(yes vs no)	1.583 (1.080–2.321)	0.018		
Moderate/poor tumor differentiation (yes vs no)	1.820 (1.295–2.557)	0.001	1.625 (1.150–2.297)	0.006
Vascular invasion (yes vs no)	2.345 (1.630–3.373)	< 0.001	1.144 (0.731–1.789)	0.557
Tumor size, cm (> 5 vs ≤ 5)	3.037 (2.104–4.384)	< 0.001	2.237 (1.130–4.429)	0.021
Multiple tumor (yes vs no)	2.917 (1.965–4.330)	< 0.001	2.021 (1.265–3.228)	0.003
Cirrhosis (yes vs no)	1.631 (1.110–2.398)	0.013		
Hypertension (yes vs no)	1.190 (0.825–1.718)	0.353		
Diabetes mellitus (yes vs no)	1.170 (0.767–1.784)	0.466		
History of smoking (yes vs no)	1.203 (0.856–1.691)	0.286		
Alcohol abuse (yes vs no)	1.215 (0.861–1.714)	0.268		
HBeAg positive (yes vs no)	1.071 (0.684–1.677)	0.765		
Child–Pugh score (B vs A)	2.224 (0.707–6.993)	0.171		
BCLC stage (B-C vs 0-A)	3.153 (2.133–4.661)	< 0.001	1.053 (0.486–2.283)	0.896
Antiviral therapy (yes vs no)	0.587 (0.418–0.825)	0.002	0.500 (0.343–0.728)	< 0.001

**Table 4:** Univariate and multivariate analysis of recurrence-free survival

	Univariate analysis		Multivariate analysis	
	HR (95% CI)	*p* value	HR (95% CI)	*p* value
Age, years (> 60 vs ≤ 60)	0.867 (0.637–1.178)	0.362		
Gender (Male vs Female)	1.127 (0.760–1.670)	0.552		
ALT, IU/ml (> 40 vs ≤ 40)	1.040 (0.769–1.406)	0.801		
AST, IU/ml (> 40 vs ≤ 40)	1.559 (1.156–2.102)	0.004		
ALP, IU/L (> 130 vs ≤ 130)	1.704 (1.240–2.340)	0.001	1.820 (1.302–2.545)	< 0.001
ALB, g/L (< 35 vs ≥ 35)	1.027 (0.697–1.515)	0.892		
Total bilirubin, µmol/L (> 17 vs ≤ 17)	1.377 (1.019–1.860)	0.037		
PT, s (≥ 13 vs < 13)	1.845 (1.249–2.725)	0.002		
AFP, ng/ml (≥ 20 vs < 20)	1.773 (1.300-2.419)	< 0.001	2.124 (1.506–2.995)	< 0.001
PLT, 10^9^/L (≥ 100 vs < 100)	1.385 (0.997–1.924)	0.052		
Satellite nodules (yes vs no)	1.956 (1.366–2.802)	< 0.001	1.290 (0.869–1.914)	0.206
Capsular (yes vs no)	1.598 (1.137–2.247)	0.007		
Moderate/poor tumor differentiation (yes vs no)	1.658 (1.226–2.242)	0.001	1.462 (1.067–2.002)	0.018
Vascular invasion (yes vs no)	2.325 (1.678–3.221)	< 0.001	1.247 (0.814–1.910)	0.311
Tumor size, cm (> 5 vs ≤ 5)	2.441(1.789–3.329)	< 0.001	1.606 (0.894–2.885)	0.113
Multiple tumor (yes vs no)	2.630(1.823–3.796)	< 0.001	1.815 (1.170–2.815)	0.008
Cirrhosis (yes vs no)	1.396 (1.009–1.933)	0.044		
Hypertension (yes vs no)	1.151 (0.830–1.596)	0.400		
Diabetes mellitus (yes vs no)	1.114 (0.762–1.627)	0.578		
History of smoking (yes vs no)	1.197 (0.887–1.616)	0.240		
Alcohol abuse (yes vs no)	1.208 (0.890–1.638)	0.225		
HBeAg positive (yes vs no)	1.110 (0.753–1.638)	0.597		
Child–Pugh score (B vs A)	2.208 (0.819–5.954)	0.118		
BCLC stage (B-C vs 0-A)	2.649 (1.908–3.678)	< 0.001	1.148 (0.591–2.231)	0.683
Antiviral therapy (yes vs no)	0.620 (0.460–0.835)	0.002	0.529 (0.377–0.741)	< 0.001

### Early and late recurrence of HCC

Of the 296 patients included in the study, 95 patients (37 in the antiviral group and 58 in the non-antiviral group) developed early tumor recurrence within 2 years after undergoing curative hepatectomy, with prothrombin time ≥ 13 s (HR, 1.954; 95% CI 1.193–3.200; *p* = 0.008), methemoglobin level ≥ 20 ng /ml (HR, 2.498; 95% CI 1.538–4.057; *p* < 0.001), platelet count ≥ 100 × 10^9^/L (HR, 1.756; 95% CI 1.087–2.838; *p* = 0.021), tumor size > 5 cm (HR, 3.035; 95% CI 1.310–7.034; *p* = 0.010), and tumor multiplicity (HR, 1.903; 95% CI 1.095–3.307; *p* = 0.023) were risk factors significantly associated with early hepatocellular carcinoma recurrence, while antiviral therapy was an independent protective factor for early recurrence (HR, 0.431; 95% CI 0.274–0.679; *p* < 0.001). Factors associated with late relapse were analyzed in 201 patients who did not develop relapse within 2 years, of whom 79 (45 in the antiviral group and 34 in the non-antiviral group) developed late relapse, and in a multifactorial analysis, AST levels > 40 IU/ml, ALP levels > 130 IU/L, and the presence of satellite nodules were independent risk factors associated with late relapse, while antiviral therapy was associated with late relapse was not associated with low risk of relapse (HR, 0.822; 95% CI 0.526–1.284; *p* = 0.389) (Tables 5, 6).

**Table 5:** Univariate and multivariate analyses for early recurrence of hepatocellular carcinoma in patients who underwent hepatic resection

	Univariate analysis		Multivariate analysis	
	HR (95%CI)	*p* value	HR (95% CI)	*p* value
Age, years (> 60 vs ≤ 60)	0.948 (0.628–1.432)	0.800		
Gender (Male vs Female)	1.246 (0.718–2.162)	0.435		
ALT, IU/ml (> 40 vs ≤ 40)	0.811 (0.533–1.234)	0.329		
AST, IU/ml (> 40 vs ≤ 40)	1.162 (0.777–1.737)	0.465		
ALP, IU/L (> 130 vs ≤ 130)	1.268 (0.812–1.980)	0.297		
ALB, g/L (< 35 vs ≥ 35)	1.249 (0.763–2.046)	0.376		
Total bilirubin, µmol/L (> 17 vs ≤ 17)	1.236 (0.824–1.855)	0.306		
PT, s (≥13 vs < 13)	2.269 (1.418–3.631)	0.001	1.954 (1.193-3.200)	0.008
AFP, ng/ml (≥20 vs < 20)	2.117 (1.361–3.291)	0.001	2.498 (1.538–4.057)	< 0.001
PLT, 10^9^/L (≥100 vs < 100)	1.648 (1.030–2.635)	0.037	1.756 (1.087–2.838)	0.021
Satellite nodules (yes vs no)	1.491 (0.910–2.442)	0.113		
Capsular (yes vs no)	1.743 (1.121–2.711)	0.014	1.565 (0.986–2.484)	0.057
Moderate/poor tumor differentiation (yes vs no)	1.812 (1.212–2.710)	0.004	1.437 (0.953–2.166)	0.084
Vascular invasion (yes vs no)	2.495 (1.643–3.788)	< 0.001	1.020 (0.605–1.722)	0.940
Tumor size, cm (> 5 vs ≤ 5)	3.898 (2.418–6.284)	< 0.001	3.035 (1.310–7.034)	0.010
Multiple tumor (yes vs no)	2.881 (1.833–4.528)	< 0.001	1.903 (1.095–3.307)	0.023
Cirrhosis (yes vs no)	1.384 (0.886–2.161)	0.154		
Hypertension (yes vs no)	0.783 (0.486–1.261)	0.315		
Diabetes mellitus (yes vs no)	1.028 (0.608–1.737)	0.918		
History of smoking (yes vs no)	1.050 (0.696–1.582)	0.817		
Alcohol abuse (yes vs no)	0.987 (0.649–1.501)	0.950		
HBeAg positive (yes vs no)	1.387 (0.847–2.272)	0.193		
Child–Pugh score (B vs A)	3.126 (0.989–9.878)	0.052		
BCLC stage (B-C vs 0-A)	4.134 (2.445–6.989)	< 0.001	1.042 (0.396–2.744)	0.933
Antiviral therapy (yes vs no)	0.488 (0.323–0.737)	0.001	0.431 (0.274–0.679)	< 0.001

**Table 6:** Univariate and multivariate analyses for late recurrence of hepatocellular carcinoma in patients who underwent hepatic resection

	Univariate analysis		Multivariate analysis	
	HR (95% CI)	*p* value	HR (95% CI)	*p* value
Age, years (> 60 vs ≤ 60)	0.777 (0.490–1.232)	0.284		
Gender (Male vs Female)	1.008 (0.574–1.769)	0.979		
ALT, IU/ml (> 40 vs ≤ 40)	1.385 (0.890–2.153)	0.149		
AST, IU/ml (> 40 vs ≤ 40)	2.229 (1.421–3.496)	< 0.001	1.835 (1.151–2.926)	0.011
ALP, IU/L (> 130 vs ≤ 130)	2.405 (1.524–3.795)	< 0.001	2.063 (1.271–3.349)	0.003
ALB, g/L(< 35 vs ≥ 35)	0.777 (0.411–1.470)	0.438		
Total bilirubin, µmol/L (> 17 vs ≤ 17)	1.565 (1.001–2.448)	0.050		
PT, s(≥13 vs < 13)	1.231 (0.592–2.559)	0.577		
AFP, ng/ml (≥20 vs < 20)	1.468 (0.940–2.292)	0.091		
PLT, 10^9^/L (≥100 vs < 100)	1.152 (0.723–1.835)	0.552		
Satellite nodules (yes vs no)	2.808 (1.665–4.737)	< 0.001	2.051 (1.174–3.584)	0.012
Capsular (yes vs no)	1.414 (0.825–2.423)	0.208		
Moderate/poor tumor differentiation (yes vs no)	1.477 (0.931–2.344)	0.098		
Vascular invasion (yes vs no)	2.086 (1.229–3.539)	0.006	1.306 (0.651–2.619)	0.453
Tumor size, cm (> 5 vs ≤ 5)	1.547 (0.990–2.416)	0.055		
Multiple tumor (yes vs no)	2.223 (1.172–4.215)	0.014	1.390 (0.656–2.945)	0.390
Cirrhosis (yes vs no)	1.411 (0.878–2.269)	0.155		
Hypertension (yes vs no)	1.750 (1.106–2.769)	0.017	1.458 (0.914–2.327)	0.114
Diabetes mellitus (yes vs no)	1.222 (0.706–2.115)	0.475		
History of smoking (yes vs no)	1.399 (0.899–2.177)	0.137		
Alcohol abuse (yes vs no)	1.534 (0.982–2.396)	0.060		
HBeAg positive (yes vs no)	0.813 (0.430–1.538)	0.525		
Child–Pugh score (B vs A)	1.173 (0.163–8.438)	0.874		
BCLC stage (B-C vs 0-A)	1.809 (1.156–2.830)	0.009	1.397 (0.832–2.345)	0.207
Antiviral therapy (yes vs no)	0.822 (0.526–1.284)	0.389		

### Cirrhosis and HBeAg subgroup analysis

**Fig. 2:**
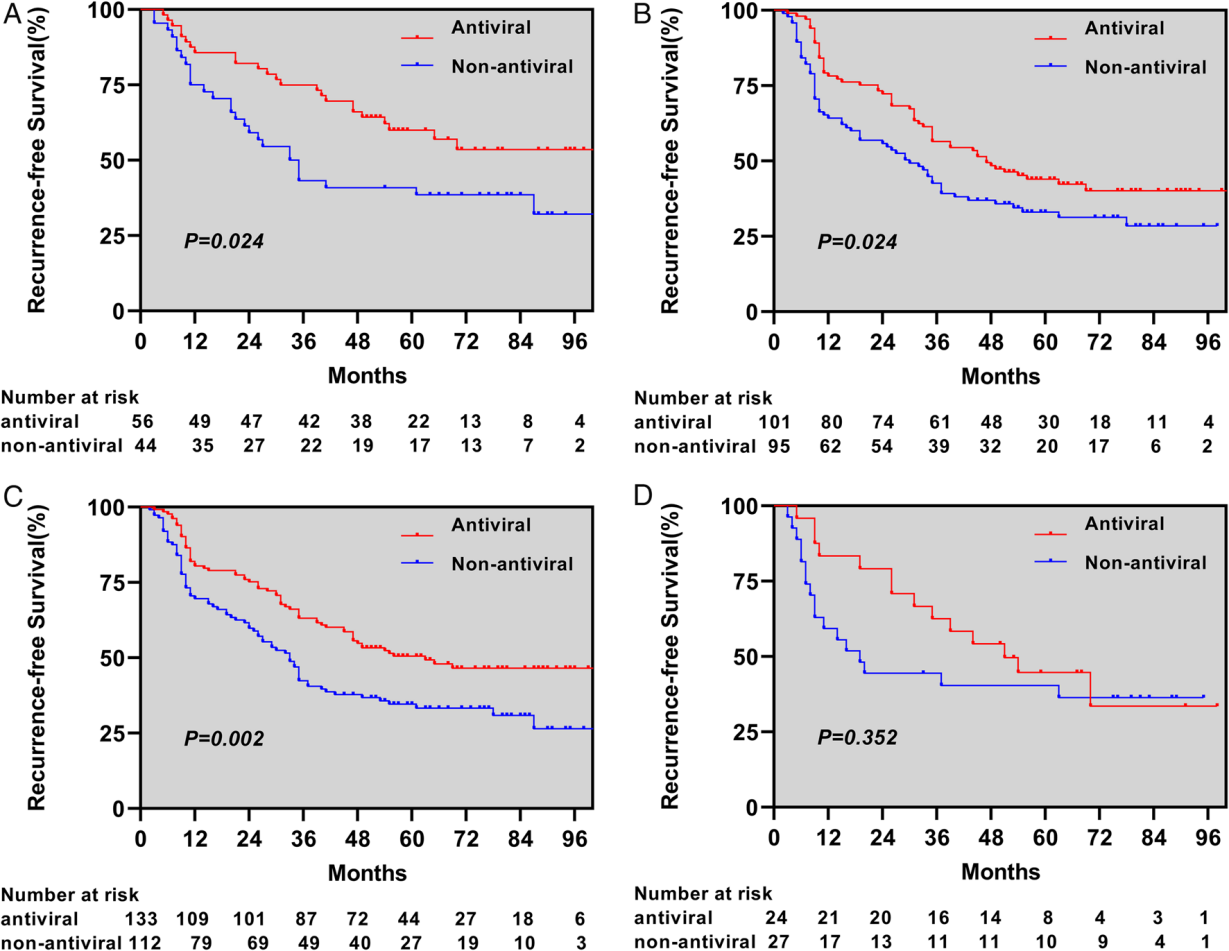
Kaplan-Meier plots of recurrence-free survival for antiviral versus non-antiviral. Patients with absence of cirrhosis (**A**) and presence of cirrhosis (**B**). Patients with absence of HBeAg (**C**) and presence of HBeAg (**D**)

Patients in both groups who received antiviral therapy or not were stratified according to cirrhosis and HBeAg levels, and the results showed that the 1-, 3-, and 5-year RFS were significantly better in the antiviral group than in the non-antiviral group, with or without cirrhosis (*p* = 0.024, *p* = 0.024), and in the HBeAg-positive group, the 1-, 3-, and 5-year RFS in the antiviral group were not significantly different from those in the non-antiviral group (*p* = 0.352), while in the HBeAg-negative group, the 1-, 3-, and 5-year RFS was significantly longer in the antiviral group than in the non-antiviral group (*p* = 0.002) (Fig. 2).

## Discussion

In this study, we included 296 patients with HBV-associated HCC who underwent hepatectomy at our institution to assess risk factors for early and late recurrence and long-term prognostic outcomes, and we found that antiviral therapy significantly improved OS, RFS, and reduced early recurrence compared with no antiviral therapy, but did not reduce the risk of late recurrence of HCC. In a subgroup analysis of cirrhosis and HBeAg, our study showed that antiviral therapy improved the prognosis of patients regardless of the presence or absence of cirrhosis and significantly prolonged RFS in HBeAg-negative patients compared to HBeAg-positive patients.

Despite the progress in the treatment of HCC in terms of surgery and neoadjuvant therapy, there is still no effective means to prevent the recurrence of HCC, and how to reduce the recurrence of HCC remains a major problem for clinicians. Persistent viral replication, chronic hepatitis activity, clinical features of the tumor, liver fibrosis, and the patient’s immune status are thought to be the main causes of recurrence after hepatectomy [5, 15, 22, 23]. In patients with HBV-associated HCC, the viral replication status, on the other hand, is a key factor influencing recurrence, and HBV-DNA levels are highly correlated with the degree of liver fibrosis and the patient’s inflammatory status, which reflects the degree of viral replication in the body [24–27]. Some studies have shown that high levels of HBV-DNA are the main risk factor affecting the long-term prognosis of patients with HBV-associated HCC after hepatectomy [28–31]. It has also been shown that low levels of HBV-DNA may also reduce mortality in patients [32]. This suggests that regardless of the level of HBV-DNA, the presence of a persistent viral replication state affects the survival status of patients, and although HBV-DNA is undetectable in most HBeAg-positive patients, viral replication in the body does not cease, and chronic inflammatory and immune damage to the liver remains, making inhibition of HBV-DNA replication particularly important. Antiviral therapy not only inhibits viral replication in the body, significantly reduces the risk of viral reactivation and improves liver function reserve, but also inhibits hepatitis activity, reduces the inflammatory response of the liver and even reverses cirrhosis [5, 33, 34]. Protein X (HBx), a key viral oncoprotein encoded by HBV, can lead to upregulation of its activity and promote tumor growth after specific ubiquitination by male-specific lethal 2 (MSL2), whereas antiviral therapy can significantly reduce HBx mRNA expression in tumor tissues and slow down tumor progression [35]. Our findings show that in HBV-associated HCC patients with low levels of HBV-DNA, preoperative or early postoperative antiviral therapy significantly improves overall survival and recurrence-free survival, as observed in the whole cohort and PSM cohort.

Better RFS may be the result of better protection of residual liver function, and antiviral therapy may enhance viral clearance after hepatectomy in patients with HBV-associated HCC, protect residual liver function, and promote hepatocyte regeneration, which in turn reduces tumor recurrence [36, 37]. Early recurrence of hepatocellular carcinoma is mostly associated with clinical features of the tumor, including invasion of the envelope, the presence of satellite nodes, vascular invasion, and tumor number and size, while chronic hepatitis activity, and immune status are considered to be associated with late recurrence [18, 38, 39]. Our study showed that multiple factors were associated with early recurrence in multivariate COX regression analysis, including prolonged prothrombin time (≥ 13s), methemoglobin level ≥ 20ng/ml, platelet count ≥ 100 × 10^9^/L, tumor size > 5 cm, multiple tumors, and not receiving antiviral therapy, while late recurrence was associated with AST levels > 40 IU/ml, ALP levels > 130 IU/L, and the presence of satellite nodules. This study also showed that antiviral therapy was an independent protective factor for early recurrence, but not for late tumor recurrence. Several recent studies have also reported that preoperative antiviral therapy significantly reduces the risk of early tumor recurrence after hepatectomy in patients with HBV-associated HCC [18, 33, 40], which differs from Huang et al. [23] who suggested that antiviral therapy had no protective effect on early tumor recurrence and reduced the incidence of late recurrence. We speculate that this discrepancy may be explained by the fact that antiviral therapy reduces the incidence of MVI after hepatectomy, which is considered a risk factor for early tumor recurrence. HBV promotes angiogenesis by enhancing the expression of metastasis-associated protein 1 and enhances the vascular metastasis of tumors by disrupting the body’s immune response against the primary tumor [41, 42]. In addition, several clinical studies have demonstrated that patients with HCC due to HBV infection are more likely to develop MVI than patients with non-HBV-associated HCC [43, 44]. These results suggest that antiviral therapy may be associated with MVI, which in turn affects the early recurrence of liver tumors. In addition, studies have shown that late recurrence of tumors has different oncological features from early recurrence and is not associated with primary HCC, which is considered as a new tumor, and that splenic stiffness measurement(SSM), a non-invasive marker for assessing portal hypertension, is an independent predictor of late recurrence of HCC. predictor, SSM predicts complications of liver disease, including carcinogenesis, and patients with SSM > 70 kPa are significantly associated with late recurrence of HCC [45]. Some studies have also suggested that men is an independent risk factor for late recurrence and tried to explain this gender difference by sex hormones, the use of estrogen reduced the occurrence of HCC, estrogen inhibits the anti-inflammatory effects of NF-κB pathway and can inhibit the release of pro-inflammatory cytokines, which in turn affects the regulation of oxidative and stress pathways in carcinogenesis [46, 47]. Naugler et al. [48] showed that estrogen treatment inhibited interleukin 6 production by blast cells in female mice, leading to reduced hepatocellular carcinogenesis. In conclusion, these results collectively illustrate the complexity of early and late tumor recurrence, which is caused by multiple factors in liver tumors, and elucidate the relevant factors affecting HCC recurrence, pending additional and more in-depth studies.

However, there are some limitations of this study. First, this is a retrospective cohort study, which is susceptible to many biasing factors, and we minimized potential bias and confounding variables by propensity score matching. Second, there was heterogeneity in the timing of initiation of antiviral therapy, with most patients starting antiviral therapy within 3 months preoperatively, which may be associated with a better prognosis and may influence HBV reactivation, and 100 patients starting antiviral therapy only within 3 months postoperatively. Third, all study subjects were from a single medical institution, although this was a large provincial tertiary care general teaching hospital, lacking a multicenter sample, and we did not perform HBV genotype testing, yet patients with HBV genotypes A and B are most common in Asia [49], which leads to a limited generalization of the experimental results. Our findings need to be further validated in additional randomized controlled trials and large multicenter studies.

In conclusion, our study showed that in HBV-associated HCC patients with low levels of HBV-DNA undergoing curative hepatectomy, tumor factors and methemoglobin levels were associated with early recurrence, liver function blood biochemical parameters determined late recurrence, and antiviral therapy reduced early tumor recurrence and improved OS, resulting in long-term survival benefits for patients. We suggest that patients with low levels of HBV-DNA should also receive antiviral therapy as early as possible.

## Data Availability

Please contact author for data requests.

## Abbreviations

HCC: Hepatocellular carcinoma
HBV: Hepatitis B virus
PSM: propensity score matching
AFP: Alpha-fetoprotein
ETV: Entecavir
TDF: Tenofovir disoproxil fumarate
HBeAg: Hepatitis B e antigen
HBx: Protein X
MSL2: Male-specific lethal 2
SSM: Splenic stiffness measurement
